# The Treatment of Psychotic Depression in a 64-Year-Old Patient With Carcinoid Syndrome

**DOI:** 10.7759/cureus.23905

**Published:** 2022-04-07

**Authors:** Jason Ng

**Affiliations:** 1 Core Psychiatry Training, South London and Maudsley National Health Service (NHS) Foundation Trust, London, GBR

**Keywords:** gastrointestinal carcinoid tumor, carcinoid syndrome, clinical psychiatry, vortioxetine, psychotic depression, psychiatry, carcinoid tumour, neuroendocrine tumour, psychiatric pharmacology

## Abstract

Serotonin-producing tumours are a subset of neuroendocrine tumours which, when active, can lead to carcinoid syndrome caused by the secretion of vasoactive substances such as serotonin, histamine, and bradykinins. In addition to the common symptoms of flushing, diarrhoea, abdominal pain, and carcinoid heart disease, carcinoid syndrome is also known to cause psychiatric symptoms of depression as well as anxiety and, very rarely, psychotic symptoms. The treatment of the neuropsychiatric manifestations of the disease is also complicated by the concurrent use of somatostatin analogues, which also affect total circulating serotonin levels, a neurotransmitter implicated in the aforementioned psychiatric disorders. In this case report, we will discuss the management of psychotic depression in a 64-year-old female patient with a metastatic carcinoid tumour and our rationale for the use of vortioxetine in her treatment.

## Introduction

Neuroendocrine tumours are a form of neoplastic disease, with an estimated rate of one per 100,000 individuals, with an almost equal ratio of tumour incidence in both male and female and a median age at diagnosis of 63 years [1]. They are commonly slow-growing, and arise in tissues that secrete hormones in the lungs or gastrointestinal tract, leading to the formation of solid malignant tumours [1]. A unique trait of neuroendocrine tumours is their ability to produce a diverse range of hormones as well as amines, including bradykinins, histamine, and prostaglandins into the body’s circulation [1]. Approximately 10% of serotonin-producing neuroendocrine tumours lead to carcinoid syndrome, which occurs when biologically active substances secreted by the tumour bypass liver metabolism either by way of the tumour not draining into the portal system or due to hepatic metastasis, and enter the bloodstream [1]. The symptoms of carcinoid syndrome arise from vasoactive substances such as serotonin and bradykinins, which commonly lead to flushing, diarrhoea, abdominal pain, and carcinoid heart disease [1].

Psychiatric symptoms have also been reported in metastatic carcinoid tumour, with a reported incidence of 50% for depression and 35% for anxiety [2]. While several case reports have documented an association between carcinoid syndrome and psychotic symptoms, the precise mechanism and subsequently treatment methods are unclear [2]. In this article, we report the case of a 64-year-old woman presenting with psychotic depression on a background of a mesenteric carcinoid tumour, according to CARE 2017 guidelines for case reports [3].

## Case presentation

Patient information

The patient in this case is a 64-year-old British Caucasian woman who was voluntarily admitted in 2021 to an inpatient psychiatric unit for symptoms of psychotic depression. In 2016, she was diagnosed with a mesenteric carcinoid tumour, with metastasis to the liver and spine. This was found during an annual assessment with her general practitioner when she was complaining of flushing, diarrhoea, and abdominal pain. She was subsequently referred to endocrinology, whereupon they confirmed the diagnosis of a serotonin-secreting neuroendocrine tumour in the midgut through the findings of high 5-HIAA urine levels, in addition to a scintigraphy method known as Octreoscan. Her care was transferred to the endocrine oncology department, where the team prescribed a somatostatin analogue, lanreotide, and a tryptophan hydroxylase inhibitor, telotristat, with the aim of decreasing total circulating serotonin and achieving symptomatic control of the carcinoid syndrome. Her symptoms improved on this regime, although she still reported the occasional episode of diarrhoea or flushing.

Her symptoms of depression started a year before her admission, after receiving news of her prognosis being five years. This began with mild-to-moderate symptoms of depression; low mood, anhedonia, reduced appetite and sleep, cognitive symptoms of reduced attention and concentration, as well as memory problems. There was no previous psychiatric history with the exception of a 20-year history of auditory pseudo-hallucinations of which the patient was aware and they did not affect her level of functioning. This was reviewed by a community psychiatrist, who determined it to be negative thoughts rather than psychotic phenomena. There was no family history of mental illness in the family and no evidence of alcohol or substance misuse. The patient is currently retired, previously having worked as a secretary, is single, and lives with her 58-year-old sister.

From the initial diagnosis of depression, the patient was started on sertraline in addition to cognitive behavioural therapy by the endocrine oncology team. This was effective for a span of three months, followed by a relapse of her depressive symptoms. Her sertraline, which was optimised to the maximum dosage of 200 mg, was still ineffective, leading to a switch to citalopram. Citalopram was started at 20 mg, and subsequently uptitrated to the max dose of 40 mg with minimal effect. This was then followed by mirtazapine, and during this period, the patient’s symptoms started to gradually worsen, and she developed psychotic symptoms of nihilistic delusions, persecutory delusions and delusions of guilt, as well as formal thought disorder, which included alogia, thought blocking, and tangential speech. She was brought into the Accident and Emergency department by her sister, and was subsequently assessed by the psychiatric team, leading to the voluntary admission of the patient to a general adult inpatient ward.

Clinical management

On admission, an ECG (electrocardiogram) was performed, showing normal sinus rhythm with QT interval within normal limits. The patient was started on 200 mg of amisulpride twice daily for treatment of her psychotic symptoms, in addition to the antidepressant she was currently on, 45 mg of mirtazapine daily. Her blood tests on admission, which included a full blood count, urea and electrolytes, thyroid function tests, liver function tests, vitamin D, vitamin B12, and folate were within normal limits. Her urine 5-HIAA levels were within normal range as well, indicating a normal level of circulating serotonin. A CT scan was performed while the patient was in Accidents and Emergency, followed by an MRI head scan. Both modalities of neuroimaging displayed no evidence of a space-occupying lesion or brain metastases.

This combination of amisulpride and mirtazapine was trialled for two weeks, which led to an improvement of her psychotic symptoms, with a resolution of her persecutory delusions and delusions of guilt. During this period, as-required lorazepam was also prescribed, with improvements in sleep pattern from three to four hours of sleep a night to seven hours of sleep. However, the patient’s depressive symptoms were still predominant, with poor intake, anhedonia, low mood, and negative beliefs around self and the future. The patient’s antidepressant medication, mirtazapine, was subsequently switched to vortioxetine 10 mg daily, which led to a rapid improvement of depressive symptoms within the span of 10 days. The patient had an improvement of appetite and cognition, increased daily activities, mood reactivity, sleep, and a more future-oriented and positive outlook on her life.

Follow-up and outcomes

**Figure 1:**
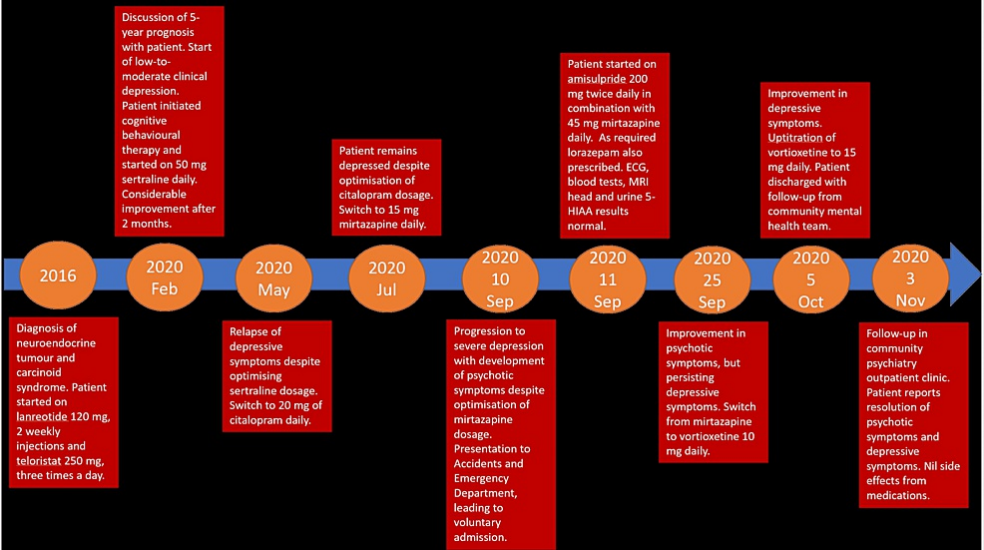
Timeline of Disease Progression, Interventions, and Outcome

Following a four-week admission, the patient was subsequently discharged on vortioxetine 15 mg daily and amisulpride 200 g twice daily with further follow-up from a affective-disorder-focused community mental health team. At the one-month follow-up in the outpatient psychiatry clinic, the patient reported that her mood had improved significantly, along with appetite, sleep and cognitive faculties returning to baseline. She is continuing to engage with cognitive behavioural therapy provided from the endocrine oncology team and while she states that she would sometimes have negative thoughts and drop in mood, feels that this is an appropriate response to having to deal with her prognosis and chronic medical condition. There were no reported side effects from the vortioxetine-amisulpride combination, including an absence of adverse serotonin-related symptoms. The timeline of disease progression, management, and outcomes is given in Figure 1.

## Discussion

While there is an association between the prevalence of anxiety and depression in patients with carcinoid syndrome, it is unclear as to whether this phenomenon can be solely attributed to emotional distress from chronic symptoms as well as the poor prognosis, or if the effects of hormone overproduction should be considered [4]. This is further complicated by the interplay of medications involved in treating comorbid depression and carcinoid syndrome, where antidepressants aim to increase serotonin levels, whereas somatostatin analogues aim to produce the opposite effect [5].

While carcinoid syndrome is caused by the over-secretion of serotonin, there appears to be a paradoxical decrease in brain serotonin production [5]. This is due to peripheral serotonin being unable to cross the blood-brain barrier, and as such, depends on the availability of serotonin’s precursor molecule, tryptophan [5]. L-tryptophan or free tryptophan can only enter the brain by crossing the blood-brain barrier, thus leading to reduced levels in the brain as the majority of the serotonin precursor molecules are consumed peripherally from the overproduction of serotonin by the carcinoid tumour [5]. L-tryptophan level is also reduced by substances like cortisol, the levels of which increase in depression, further reducing serotonin production in the brain [6].

In this particular case, conventional antidepressants such as selective serotonin reuptake inhibitors (SSRIs) and noradrenergic and specific serotonergic antidepressant (NaSSA) were used for an adequate duration and dosage, with minimal effect. According to the National Institute for Health and Care Excellence (NICE) guidelines for the treatment of depression, the patient’s progressively declining physical health and lack of response to three trials of antidepressants would necessitate the consideration of electroconvulsive therapy (ECT) [7]. However, on further discussion among the managing team as well as the endocrine oncologists on the possible pathophysiology of her current illness, we opted for a trial of vortioxetine.

From this knowledge, we hypothesised that the reason for the ineffectiveness of the antidepressant was due to the lack of serotonin production, in which case the increase of serotonin reuptake would lead to minimal changes in central serotonin levels. Vortioxetine has a unique mode of action in comparison to other antidepressants, in that it is a direct partial agonist of 5-HT1, the main serotonin receptor thought to be related to depression [8]. This proved to be an effective treatment, leading to a significant improvement of both psychotic and depressive symptoms.

While comorbid depression as well as anxiety in patients with carcinoid syndrome have been well-characterised, there have been only three case reports of psychotic symptoms in patients with carcinoid syndrome [9]. Interestingly, 2 out of 3 cases involved patients that had an absence of past psychiatric history, similar to the current case. The use of vortioxetine in depressed patients with carcinoid syndrome has also not been well-characterised, and has been mentioned mainly in drug safety studies [8].

We hope this case report would be helpful in adding to the literature on psychotic depression in carcinoid syndrome, as well as its possible treatment modalities. Admittedly, this report would have been more beneficial with the use of psychotic and depressive rating scales, such as the Positive and Negative Syndrome Scale (PANSS) and Beck’s Depression Inventory (BDI), in order to better monitor clinical improvements and provide a more direct comparison in symptomatology. Additionally, the lack of ability to directly measure serotonin levels in the brain has also made it difficult to identify the specific cause of the patient’s psychotic depression [10].

With that said, the rapid improvement of the patient on vortioxetine suggests that serotoninergic brain dysfunction might be a possible mechanism for psychosis and depression in patients with carcinoid syndrome. To date, there have been few prospective studies researching the clinical features of psychiatric disorders in patients with neuroendocrine tumours, and this is an area that we think is more deserving of attention. Furthermore, cases such as this one also highlight the importance of a multidisciplinary approach to the management of neuropsychiatric manifestations of oncological disorders, as well as an awareness and appreciation of the neurophysiology that underpins the disease.

## Conclusions

Going through the current medical literature, there is limited research on the treatment of psychotic depression in carcinoid syndrome, as well as the complex interactions between the changes in serotonin levels brought on by the carcinoid tumour, antidepressants, and antipsychotic use. This case report adds to the existing literature by supporting the potential associations between serotonergic brain dysfunction, psychiatric symptom manifestation, and carcinoid syndrome. It also highlights the importance of an awareness of the cross-over between endocrine-oncology and psychiatry, in addition to having a better understanding of the neurobiology of organic illnesses. We hope that in the future, further developments in functional neuroimaging would enable us to better understand these phenomena and their role in the treatment and management of neurotransmitter-related pathology.
